# NMR Investigation of the Interaction of Three Non-Steroidal Anti-Inflammatory Drugs with Human Serum Albumin

**DOI:** 10.3390/molecules27196647

**Published:** 2022-10-06

**Authors:** Federica Aiello, Gloria Uccello-Barretta, Claudio Picchi, Samuele Nazzi, Alessandra Recchimurzo, Federica Balzano

**Affiliations:** 1 National Research Council, Institute for Chemical and Physical Processes (CNR-IPCF), Via G. Moruzzi 1, 56124 Pisa, Italy; 2 Department of Chemistry and Industrial Chemistry, University of Pisa, Via G. Moruzzi 13, 56124 Pisa, Italy

**Keywords:** protein/molecule interaction, NSAIDs, selective relaxation rates, affinity index, binding, NMR

## Abstract

The understanding of the interaction between non-steroidal anti-inflammatory drugs and human serum albumin plays a fundamental role in the development of new drugs and new therapeutic strategies. Several studies have been performed, nevertheless, the interaction phenomena are still not fully understood. In this work, high-field solution Nuclear Magnetic Resonance (NMR) spectroscopy was applied to compare the strength of the interaction of diclofenac sodium salt, ketorolac tris salt and flurbiprofen sodium salt toward albumin. To this aim, mono- and bi-selective relaxation rate measurements were performed by applying selective π-pulses at the selected frequencies and by following magnetization recovery. On the basis of the dependence of relaxation parameters on albumin concentration, normalized affinity indexes were calculated for several protons of the drugs. Affinity indexes for diclofenac were about five-fold higher in comparison with ketorolac and flurbiprofen. Aromatic moieties of the three drugs and methine protons at the chiral centers of ketorolac and flurbiprofen were more involved in the interaction with albumin. In conclusion, NMR spectroscopy allows not only for the comparison of drug-to-protein affinities but also points out the nature of the drug sites that are more extensively involved in the interaction.

## 1. Introduction

Nowadays non-steroidal anti-inflammatory drugs (NSAIDs) are among the most commonly used pharmaceuticals. These compounds are used for the treatment of several conditions in virtue of their anti-inflammatory, analgesic, antipyretic, and anti-aggregant properties [1]. Depending on the nature of the drug, oral, intraocular, intravenous, intramuscular, topical and rectal administration can be selected. In all cases, the drug reaches the site of action through the bloodstream, interacting with the proteins present in the blood plasma, especially human serum albumin (HSA), a globular protein that accounts for 60% of the total protein content. Efficient drug delivery systems, such as albumin-based nanoparticles [2], were proposed as well.

The remarkable flexibility together with the high concentration in the blood and the presence of multiple binding sites make HSA particularly inclined to interact with small molecules such as anticoagulants, anesthetics, steroids, amino acids and, indeed, NSAIDs [3]. The strength of this interaction has a direct effect on the bioavailability and on the metabolism: if the interaction with HSA is too strong, the drug cannot be released in the body, or a higher concentration is necessary to guarantee therapeutic efficacy. On the other hand, if the interaction is too weak the drug could be metabolized before reaching the site of action. In spite of the fact that the knowledge of the drug-to-HSA interaction mechanisms is fundamental for optimizing pharmacokinetic, pharmacodynamic, and toxicological profiles, a profound and complete comprehension of binding phenomena is still far from being achieved.

Several studies were performed, starting in the 1980s [4,5,6], to understand and rationalize the nature of the interactions between NSAIDs and HSA. Different analytical techniques were exploited to reach this goal: for example, isothermal titration calorimetry was combined with frontal analysis/capillary electrophoresis to shed light on the thermodynamic parameters of the interaction [7]. Fluorescence spectroscopy was employed for detecting the formation of the drug/albumin complex and obtaining the binding parameters [8]. Molecular modeling and spectroscopic techniques (fluorescence and UV) contributed to improving the comprehension of the interaction of HSA with diclofenac sodium salt, highlighting the pivotal role played by the hydrophobic interactions in the binding, with hydrogen bonds acting as support [9]. Such interactions were confirmed also via X-ray crystallography, which pointed out the protein residues involved in the interaction with the drug [10]. Competitive binding processes were investigated through circular dichroism and fluorescence spectroscopy [11] and better mastery of more complex binding processes, e.g., photo-induced ones, was obtained by UV spectroscopy [12].

**Scheme 1:**
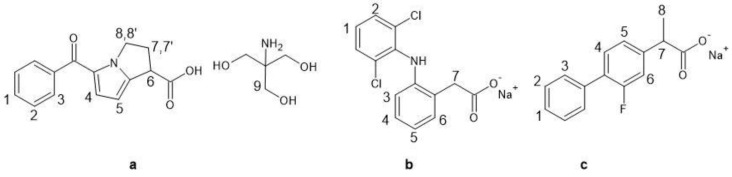
Chemical structures and numbering schemes for NMR analysis of (**a**) KTR, (**b**) DCF and (**c**) FBP.

NMR constitutes an analytical technique largely employed in the investigation of drug/biomacromolecule interactions [13,14]. As a matter of fact, NMR spectroscopy allows determining not only the complexation stoichiometry and the association constants or, more generally, the affinity of the drug for the protein, but also the stereochemical parameters, thus providing information about the nature of the sites more extensively involved in the interaction. In this work, an NMR investigation was performed on the interaction between HSA and three drugs belonging to the group of arylalcanoic acid derivatives that are among the most used NSAIDs, known for their antipyretic and anti-inflammatory properties: ketorolac tris salt (KTR), diclofenac sodium salt (DCF) and flurbiprofen sodium salt (FBP), reported in Scheme 1. The interaction was investigated by measuring the mono-selective spin-lattice relaxation rates (R_1_^ms^) of KTR, DCF and FBP nuclei in the presence of HSA since this parameter is much more sensitive to the occurrence of interactions compared to the non-selective relaxation rate (R_1_^ns^) [15].

The interaction between KTR and HSA has already been studied mainly via chromatographic methods [16] and, to the best of our knowledge, has not been investigated in detail via NMR, whereas there is only one NMR study focused on the low-affinity interaction between HSA and DCF, based on the analysis of chemical shift and linewidth of the ^13^C NMR signals of the drug in the presence of the protein [17]. Regarding FBP, its interaction with HSA was followed via NMR diffusion measurements, which were used for the calculation of the ligand–protein dissociation constant and the stoichiometry of binding [18]. The presence of a fluorine nucleus on the skeleton, moreover, made possible the use of ^19^F NMR spectroscopy as an investigation tool [19].

## 2. Results and Discussion

Proton mono-selective relaxation rates (R_1_^ms^) of KTR, DCF and FBP in their free state and in a mixture with HSA were calculated from the corresponding mono-selective relaxation times (T_1_^ms^) measured for each drug in D_2_O (pH 7.4, phosphate buffer, 0.1 M), according to Equation (1).

R_1_^ms^ = 1/T_1_^ms^ (1)

This parameter was chosen as an interaction probe taking into consideration the experimental conditions in which the studies were carried out. The notable difference in the molecular weight of the species involved, in fact, does not allow working with solutions at the equimolar ratio, since the NMR signals of the small molecule (the drug) would be hardly superimposed on those belonging to the macromolecule. Therefore, it is necessary to exploit a parameter highly sensitive to the interaction even when the macromolecule is in strong defect with respect to the drug, such as the mono-selective relaxation rates.

It is important to underline that, rather than R_1_^ms^, the corresponding normalized relaxation rate (ΔR/R_f_, where ΔR = R_obs_ − R_f_) constitutes a very efficient tool for comparing the entity of the interaction of different protons of the drug. The determination of R_1_^ms^ of the drugs’ nuclei in mixtures containing different amounts of HSA allows extracting their normalized affinity indexes ([A^N^]), a measure of the drug–HSA global affinity and a useful parameter to compare the ability of different ligands to interact with the same macromolecule.

For protons at a fixed and known distance, the cross-relaxation terms (σ) can be obtained as the difference between bi- and mono-selective relaxation rates, allowing the bound molar fractions (x_b_) to be calculated, as discussed in Appendix A [15,20,21].

### 2.1. KTR/HSA Mixtures

No superimposition was observed for KTR nuclei in the proton spectrum (Figure S1), hence it was possible to measure the relaxation parameters for almost all nuclei. All KTR protons increased their relaxation rate with an increasing HSA concentration, as reported in Table S1. The normalized values, reported in Table 1, indicate that the protons belonging to the two aromatic moieties (H_1_ and H_4_/H_5_) and the proton in α-position with respect to the carboxylate group (H_6_) are highly involved in the interaction. As an example, proton H_1_ undergoes more than a ten-fold increase in its mono-selective relaxation rate, which goes from 0.26 s^−1^ in the free state to 3.03 s^−1^ (Table S1) in the presence of the highest amount of protein (2 mg/mL).

**Figure 1:**
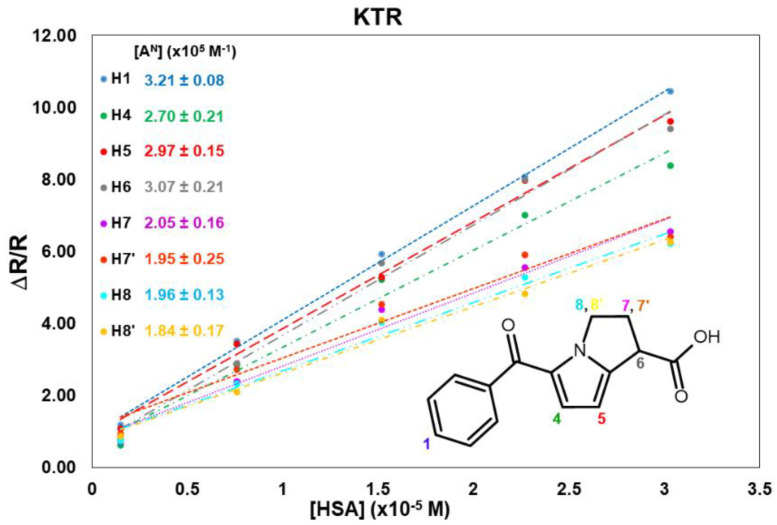
Normalized mono-selective relaxation rates plotted vs. HSA concentration and calculated affinity indexes for KTR protons.

The linear fitting of normalized mono-selective relaxation rates vs. albumin concentration allowed calculating [A^N^] (see Appendix A) for KTR nuclei (Figure 1), and values of normalized affinity indexes ranging from 1.8 × 10^5^ M^−1^ to 3.2 × 10^5^ M^−1^ were obtained (see also Table S2 in Supplementary Material). Data confirm that the aromatic protons H_1_ and H_4_/H_5_ and proton H_6_, directly linked to the carboxyl group, are the nuclei more influenced by the protein.

By simultaneous inversion of aromatic protons H_4_ and H_5_, at a known and fixed distance, the bi-selective relaxation rate R^bs^_4-5_ was measured to obtain the observed cross-relaxation rate σ_4-5_ (σ_4-5_ = R_4-5_^bs^ − R_4_^ms^), listed in Table 1. The parameter assumes a positive value (0.05 s^−1^) for the free drug, as expected on the basis of its characteristics of motion, whereas the interaction with the protein determines a shift to negative values, typical of the slow motion region, where macromolecules and their complexes lie. The cross-relaxation term is highly sensitive to the variation of drug/protein molar ratio; in fact, it changes its values from 0 s^−1^ (KTR/HSA 1315:1) to −0.29 s^−1^(KTR/HSA 66:1).

On assuming that the reorientation time (τ_c_) of the complex is controlled by albumin (τ_c_^HSA^ = 20 ns) [22], and by keeping in mind the distance r_4-5_ (2.61 Å) [23], a value of −3.69 s^−1^ is calculated for σ_b_ on the basis of Equation (A5) (Appendix A). Once σ_obs_, σ_f_ and σ_b_ were obtained, the molar fraction in the bound state for each mixture KTR/HSA can be calculated from Equation (A6) (Appendix A). In the mixture with the higher KTR/HSA ratio (1315:1), about 1% of the drug is bound to the albumin and this amount slightly increases with decreasing of the molar ratio (9% for the mixture 66:1).

The plot of the normalized cross-relaxation terms in the function of the protein concentration gave a global normalized affinity index of 1.8 × 10^5^ M^−1^ for KTR (the slope of the line in Figure S4 in Supplementary Material).

### 2.2. DCF/HSA Mixtures

Greater variations in comparison to KTR were obtained for the aromatic nuclei of DCF in the presence of HSA (from a 17- to a 28-fold increase). The greatest variation was observed for H_2_, belonging to the chlorine-substituted aromatic ring: its mono-selective relaxation rate, equal to 0.17 s^−1^ in the free state, already increases up to 0.57 s^−1^ with a small amount of protein (0.1 mg/mL), and it remarkably undergoes almost a 29-fold increase in the mixture at the lowest molar ratio (66:1, Table 2 and Table S3 in Supplementary Material). A similar increment in the relaxation parameter is observed for the other aromatic proton H_3_.

H_5_ and H_6_, belonging to the same aromatic ring of H_3_, are strongly involved in the interaction as well. Contrary to what was observed for KTR, methylene protons (H_7_) in the α-position to the carboxyl group showed the lowest effect (Table 2).

**Figure 2:**
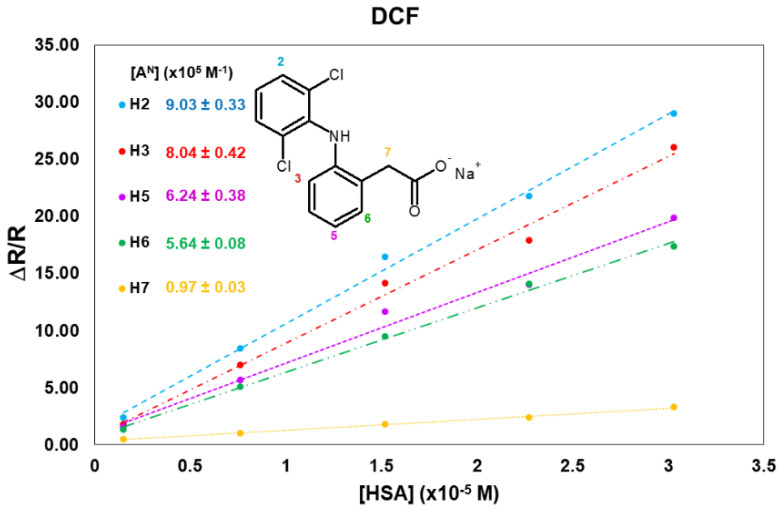
Normalized mono-selective relaxation rates plotted vs. HSA concentration, and calculated affinity indexes for DCF protons.

The normalized affinity indexes, obtained as the slope of the linear fittings shown in Figure 2, resulted very high and remarkably differentiated for DCF protons (Table S4), highlighting the differences in the binding of different groups of DCF with the HSA sites. This result is in agreement with the data obtained via fluorescence and crystallography measurements [9,10], where the important role of the hydrophobic interactions on the binding to the protein was pointed out.

Similarly to KTR, aromatic protons H_5_ and H_6_, at a known and fixed distance (2.49 Å) [24], were simultaneously inverted to obtain R_5-6_^bs^ and the corresponding cross-relaxation term (σ_5-6_). On the basis of Equation (A5) (Appendix A), a bound cross-relaxation term σ_b_ equal to −5.53 s^−1^ was obtained by assuming τ_c_ of the complex equal to τ_c_^HSA^ = 20 ns. As already discussed for KTR, the bound molar fractions at different molar ratios can be calculated from Equation (A6) (Appendix A) once σ_obs_, σ_f_ and σ_b_ are given. The comparison of the calculated x_b_ for DCF and KTR protons (Table 1 and Table 2, respectively) pointed out that the molar fraction of the two drugs is small and comparable (1% for KTR and 2% for DCF) in the solution with the lowest amount of protein (0.1 mg/mL), probably because the concentration of HSA is too low to observe significant differences in their activity. However, with the increase in the concentration of the protein, KTR and DCF show a very different affinity for the albumin, and this difference increases when increasing the protein concentration. At the lowest molar fraction, when the HSA concentration reaches 2 mg/mL, 9% of KTR is bound, in contrast to 29% of DCF.

The affinity index, calculated on the basis of the cross-relaxation term (Figure S5 in Supplementary Materials), is equal to 9.2 × 10^5^ M^−1^, five times higher than that measured for KTR.

### 2.3. FBP/HSA Mixtures

Unfortunately, the extensive superimposition between the aromatic resonances (Figure S3) did not allow for the implementation of cross-relaxation measurements for any proton pair of the drug. The mono-selective relaxation measurements performed pointed out remarkable differences in the involvement of different FBP nuclei in their interaction with HSA (Table 3 and Table S5), highlighting once again the primary role of the aromatic moiety of the drugs on the binding with HSA. While very small variations are observed for the methyl group of FBP, the relaxation rates of the aromatic proton H_3_ and methine proton (H_7_) in the α-position with respect to the carboxylate increase to a similar extent to what was observed for KTR.

**Figure 3:**
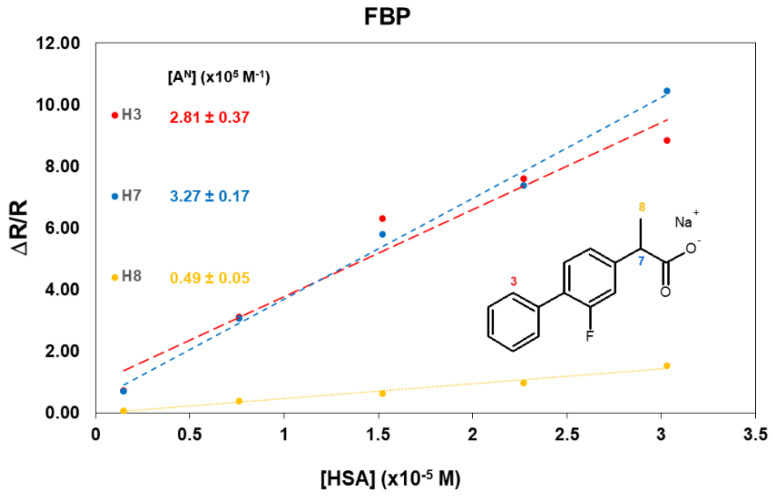
Normalized mono-selective relaxation rates plotted vs. HSA concentration, and calculated affinity indexes for FBP protons.

The different behavior of the methyl group is more evident in the mixture at the lowest molar ratio (66:1), where a value of 3.45 s^−1^ was measured for H_8_ (Table S3), corresponding to a moderate increase (normalized relaxation rate of 1.59 in Table 3), in contrast to an average ten-fold increase observed for protons H_3_ and H_7_ (Table 3). The affinity indexes calculated (Figure 3) confirm the involvement of the aromatic protons and the carboxyl group of FBP in the interaction with HSA.

## 3. Materials and Methods

### 3.1. Materials

Ketorolac tris salt (≥99%), diclofenac sodium salt (≥98%), flurbiprofen sodium salt (pharmaceutical secondary standard), human serum albumin (66 kDa) and phosphate buffer powder, 0.1 M were purchased from Sigma Aldrich (St. Louis, MI, USA). Deuterated water (D_2_O, 99.9%) was purchased from Deutero GmbH (Kastellaun, Germany). All chemical compounds were used without any further purification.

### 3.2. NMR Measurements

The NMR experiments were carried out on a Varian INOVA600 spectrometer equipped with a 5 mm probe operating at 600 MHz for ^1^H nuclei; the temperature was controlled to ±0.1 °C.

Samples were degassed to remove paramagnetic dissolved oxygen. Degassing was carried out using five freeze–pump–thaw cycles.

The spin-lattice mono-selective relaxation times (T_1_^ms^) were measured by using the inversion recovery pulse sequence (180°-τ-90°-t)_n_ and by applying a selective π-pulse at the selected frequency. Measurements were carried out for selected protons belonging to KTR, DCF and FBP, in the free state and in the presence of HSA; the drug concentration was kept equal to 2 mM for all three drugs, whereas the HSA concentration was increased from 0.1 mg/mL to 2 mg/mL.

The experimental error in the relaxation rate measurements was 5%. The errors relative to affinity indexes are reported in the Supplementary Material (Tables S2, S4 and S6).

In Appendix A, the theoretical discussion of the NMR approach with respect to mono-selective relaxation rates, cross-relaxation terms, and affinity indexes is reported.

### 3.3. NMR Characterization Data (Numbering Scheme Referred to Scheme 1)

KTR- ^1^H NMR (600 MHz, 2 mM, D_2_O pH 7.4 (0.1 mM), 298 K), δ (ppm) referred to the residual solvent (4.64 ppm): 7.64 (d, ^3^J_3-2_ = 7.6 Hz, 2H, H_3_), 7.52 (t, ^3^J_1-2_ = 7.6 Hz, 1H, H_1_), 7.42 (t, ^3^J_2-1_ = ^3^J_2-3_ = 7.6 Hz, 2H, H_2_), 6.85 (d, ^3^J_4-5_ = 4.2 Hz, 1H, H_4_), 6.03 (d, ^3^J_5-4_ = 4.2 Hz, 1H, H_5_), 4.32 (m, 1H, H_8_), 4.18 (m, 1H, H_8′_), 3.83 (dd, ^3^J_6-7_ = 8.9 Hz, ^3^J_6-7′_ = 6.0 Hz, 1H, H_6_), 3.57 (s, 6H, H_9_), 2.71 (m, 1H, H_7_), 2.57 (m, 1H, H_7′_).

DCF- ^1^H NMR (600 MHz, 2 mM, D_2_O pH 7.4 (0.1 mM), 298 K), δ (ppm) referred to the residual solvent (4.64 ppm): 7.35 (d, ^3^J_2-1_ = 8.2 Hz, 2H, H_2_), 7.14 (dd, ^3^J_6-5_ = 7.4 Hz, ^4^J_6-4_ = 1.3 Hz, 1H, H_6_), 7.03 (t, ^3^J_1-2_ = 8.2 Hz, 1H, H_1_), 6.99 (dt, ^3^J_4-5_ = ^3^J_4-3_ = 7.4 Hz, J^4^_4-6_ = 1.4 Hz, 1H, H_4_), 6.84 (dt, ^3^J_5-6_ = ^3^J_5-4_ = 7.4 Hz, ^4^J_5-3_ = 1.3 Hz, 1H, H_5_), 6.35 (dd, ^3^J_3-4_ = 7.4 Hz, ^4^J_3-5_ = 1.3 Hz, 1H, H_3_), 3.53 (s, 2H, H_7_).

FBP- ^1^H NMR (600 MHz, 2 mM, D_2_O pH 7.4 (0.1 mM), 298 K), δ (ppm) referred to the residual solvent (4.64 ppm): 7.49 (d, ^3^J_3-2_ = 7.9 Hz, 2H, H_3_), 7.39 (t, ^3^J_2-1_ = ^3^J_2-3_ = 7.9 Hz, 2H, H_2_), 7.34 (t, ^3^J_4-5_ = ^4^J_4-F_ = 8.0 Hz, 1H, H_4_), 7.32 (t, ^3^J_1-2_ = 7.9 Hz, 1H, H_1_), 7.09 (dd, ^3^J_5-4_ = 8.0 Hz, ^4^J_5-6_ = 1.4 Hz, 1H, H_5_), 7.05 (dd, ^3^J_6-F_ = 12.2 Hz, ^4^J_6-5_ = 1.4 Hz, 1H, H_6_), 3.55 (q, ^3^J_7-8_ = 7.2 Hz, 1H, H_7_), 1.29 (d, ^3^J_8-7_ = 7.2 Hz, 3H, H_8_).

## 4. Conclusions

The affinity of ketorolac tris salt, diclofenac sodium salt and flurbiprofen sodium salt towards human serum albumin was evaluated via mono-selective relaxation rate measurements, thus upholding their reliability for a detailed investigation of drug/protein interactions. The exploitation of this NMR parameter, in fact, allows for identifying the moieties of the small molecule more extensively affected by the presence of the biomacromolecule, thus leading to reliable hypotheses about the interaction mechanisms that take place in solution and offering a tool for the design of tailored drugs.

A stronger affinity of DCF for HSA with respect to KTR and FBP, which behave similarly, was highlighted. The leading involvement of the aromatic moieties was clearly pointed out for all the analyzed compounds; in particular, in the case of DCF, the aromatic ring with the chlorine atoms is more affected by the interaction compared to the other one. No significant differences between the two aromatic groups of KTR were observed, whereas the strong superimposition observed for FBP resonances did not allow the analysis of the F-bearing ring, thus preventing the comparison between the aromatic moieties.

Bi-selective relaxation measurements, associated with the mono-selective ones, allowed for the determination of the cross-relaxation rates and of the bound molar fractions for KTR and DCF; the global affinity of the two drugs for HSA, calculated from the cross-relaxation terms in the bound state, confirmed that DCF interacts more strongly with the protein, with its affinity index being five times higher with respect to KTR.

## Figures and Tables

**Table 1:** ^1^H mono-selective relaxation rates R_1_^ms^ (s^−1^) (600 MHz, 25 °C, D_2_O, pH = 7.4) of some protons of KTR (2 mM) in its free state; normalized mono-selective relaxation rates (ΔR_1_^ms^/R_f_, ΔR_1_^ms^ = R_obs_ − R_f_) of KTR protons in mixtures with HSA at different molar ratios; cross-relaxation rate (σ_ij_, s^−1^) calculated for proton pair H_4_-H_5_ and corresponding bound molar fraction (x_b_).

[HSA] mg/mL	KTR:HSA	H_1_	H_4_	H_5_	H_6_	H_7_	H_7′_	H_8_	H_8′_	σ_4-5_	x_b_
0	/	0.26	0.31	0.21	0.28	0.81	0.74	0.79	0.92	0.05	
0.1	1315:1	1.18	0.62	1.08	0.79	0.73	0.93	0.75	0.88	0.00	0.01
0.5	264:1	3.49	2.85	3.44	2.89	2.42	2.75	2.32	2.11	−0.11	0.04
1.0	132:1	5.95	5.17	5.28	5.68	4.35	4.63	4.04	4.19	−0.19	0.07
1.5	88:1	8.10	7.00	7.89	8.08	5.47	5.90	5.30	4.74	−0.23	0.08
2.0	66:1	10.58	8.34	9.70	9.41	6.69	6.50	6.41	6.27	−0.29	0.09

**Table 2:** ^1^H mono-selective relaxation rates R_1_^ms^ (s^−1^) (600 MHz, 25 °C, D_2_O, pH = 7.4) of some protons of DCF (2 mM) in its free state; normalized mono-selective relaxation rates (ΔR_1_^ms^/R_f_, ΔR_1_^ms^ = R_obs_ − R_f_) of DCF protons in mixtures with HSA at different molar ratios; cross-relaxation rate (σ_ij_, s^−1^) calculated for proton pair H_6_-H_5_ and corresponding bound molar fraction (x_b_).

[HSA] mg/mL	DCF:HSA	H_2_	H_3_	H_5_	H_6_	H_7_	σ_6-5_	x_b_
0	/	0.17	0.24	0.34	0.38	1.22	0.04	
0.1	1315:1	2.36	1.79	1.50	1.35	0.49	−0.05	0.02
0.5	264:1	8.38	6.85	5.64	5.07	1.05	−0.36	0.07
1.0	132:1	16.38	14.14	11.70	9.44	1.83	−0.77	0.15
1.5	88:1	21.73	17.90	14.37	13.50	2.42	−1.01	0.19
2.0	66:1	28.55	25.50	19.86	17.64	3.32	−1.59	0.29

**Table 3:** ^1^H mono-selective relaxation rates R_1_^ms^ (s^−1^) (600 MHz, 25 °C, D_2_O, pH = 7.4) of some protons of FBP (2 mM) in its free state and normalized mono-selective relaxation rates (ΔR_1_^ms^/R_f_, ΔR_1_^ms^ = R_obs_ − R_f_) of FBP protons in mixtures with HSA at different molar ratios.

[HSA] mg/mL	FBP:HSA	H_3_	H_7_	H_8_
0	/	0.34	0.34	1.33
0.1	1315:1	0.71	0.70	0.09
0.5	264:1	3.06	3.08	0.42
1.0	132:1	6.30	5.77	0.67
1.5	88:1	7.59	7.38	0.97
2.0	66:1	8.73	10.46	1.59

## Data Availability

Not applicable.

## Supplementary Materials

The following supporting information can be downloaded at: https://www.mdpi.com/article/10.3390/molecules27196647/s1, Figure S1: ^1^H NMR (600 MHz, D_2_O, pH 7.4, 298 K) spectrum of KTR (2 mM); Figure S2: ^1^H NMR (600 MHz, D_2_O, pH 7.4, 298 K) spectrum of DCF (2 mM); Figure S3: ^1^H NMR (600 MHz, D_2_O, pH 7.4, 298 K) spectrum of FBP (2 mM); Table S1: ^1^H mono-selective relaxation rates (ΔR_1_^ms^/R_f_, ΔR_1_^ms^ = R_obs_ − R_f_) (600 MHz, 25 °C, D_2_O, pH = 7.4) of some protons of KTR (2 mM) alone and in mixtures with HSA at different molar ratios; Table S2- Affinity indexes ([A^N^], M^−1^) and corresponding standard errors (ε, M-1) calculated for KTR protons from NMR data; Figure S4: Δσ/σ_f_ of KTR (2 mM) plotted in function of HSA concentration; Table S3: ^1^H mono-selective relaxation rates (ΔR_1_^ms^/R_f_, ΔR_1_^ms^ = R_obs_ − R_f_) (600 MHz, 25 °C, D_2_O, pH = 7.4) of some protons of DCF (2 mM) alone and in mixtures with HSA at different molar ratios; Table S4- Affinity indexes ([A^N^], M^−1^) and corresponding standard errors (ε, M-1) calculated for DCF protons from NMR data; Figure S5: Δσ/σ_f_ of DCF (2 mM) plotted in function of HSA concentration; Table S5: ^1^H mono-selective relaxation rates (ΔR_1_^ms^/R_f_, ΔR_1_^ms^ = R_obs_ − R_f_) (600 MHz, 25 °C, D_2_O, pH = 7.4) of some protons of FBP (2 mM) alone and in mixtures with HSA at different molar ratios; Table S6- Affinity indexes ([A^N^], M^−1^) and corresponding standard errors (ε, M^−1^) calculated for FBP protons from NMR data.

## Sample Availability

Samples of the compounds are commercially available.

## Appendix A. ^1^H Mono-Selective Relaxation Rates, Cross-Relaxation Terms, and Affinity Indexes

In the fast exchange regime, any NMR parameter (P_obs_) is the weighted average of its value in the free (P_f_) and bound state (P_b_), according to Equation (A1):

P_obs_ = P_f_x_f_ + P_b_x_b_ (A1)

where x_f_ and x_b_ are the molar fractions in the free and bound state, respectively.

When a drug interacts with a protein, the dynamics of the complex are driven by the latter, and it is possible to assume that the reorientation time of the complex (τ_c_) corresponds to that of the macromolecule. As a consequence of the interaction, the drug goes from the fast motion region (ω^2^τ_c_^2^ « 0.6; ω = Larmor frequency), characteristics of small molecules in their free state, to the slow-motion region (ω^2^τ_c_^2^ » 0.6) where macromolecules and their complexes are found. The non-selective relaxation parameter R_1_^ns^ increases with the increase in ω^2^τ_c_^2^ up to 0.6, and then it reaches a maximum and starts decreasing (Equation (A2)); on the contrary, the function R_1_^ms^ has a point of flex in correspondence of 0.6, and then keeps increasing with increasing of ω^2^τ_c_^2^ (Equation (A3)) [25,26,27]. This high sensitivity to the change of motion regime explains why R_1_^ms^ is usually preferred for investigations requiring a strong excess of drug with respect to the macromolecule [28].

R_1_^ns^ = 0.1 (γ^4^ħ^2^r_ij_^−6^) [3τ_c_/(1 + ω^2^τ_c_^2^) + 12τ_c_/(1 + 4 ω^2^τ_c_^2^)] (A2)

R_1_^ms^ = 0.1 (γ^4^ħ^2^r_ij_^−6^) [3τ_c_/(1 + ω^2^τ_c_^2^) + 6τ_c_/(1 + 4 ω^2^τ_c_^2^) + τ_c_] (A3)

where γ is the gyromagnetic ratio and ħ is the reduced Plank’s constant. If two nuclei ij are simultaneously inverted, it is possible to measure the bi-selective relaxation rate R_1_^bs^; the difference between R_1_^bs^ and R_1_^ms^ gives the cross-relaxation term σ_ij_, a parameter that is dependent on the τ_c_ of the drug and from the proton distance r_ij_. For small molecules in the fast-motion region, σ_ij_ can be calculated according to Equation (A4), whereas for macromolecules and their complexes one must refer to Equation (A5).

σ_ij_ = 0.5γ^4^ħ^2^r_ij_^−6^τ_c_ (A4)

σ_ij_ = −0.1γ^4^ħ^2^r_ij_^−6^τ_c_ (A5)

In the case of a drug/protein complex, Equation (A5) can be used for calculating the bound cross-relaxation term (σ_b_) for a proton pair at a known distance, when τ_c_ is known for the protein. Considering that a large excess of the ligand is used with respect to the macromolecule, the free molar fraction of the drug can be approximated to 1, and, from Equation (A1), the bound molar fraction of the drug can be obtained (Equation (A6)).

x_b_ = (σ_obs_ − σ_f_)/σ_b_ (A6)

The extent to which different drug nuclei are involved in the interaction with the protein can be better evaluated by calculating the normalized mono-selective relaxation rate (Equation (A7)).

∆R/R_f_ = (R_obs_ − R_f_)/R_f_ (A7)

It is then possible to rewrite Equation (A1) in the form of Equation (A8), approximating the molar fraction of the free ligand to 1.

R_obs_ = R_f_ + R_b_x_b_ (A8)

In the occurrence of a 1:1 complexation equilibrium between the drug (L) and the macromolecule (M), the hetero association constant K can be expressed according to Equation (A9):

K = [ML]/(([M_0_] − [ML])[L]) (A9)

where [ML] is the concentration of the drug/macromolecule complex, [M_0_] is the initial concentration of the macromolecule and [L] is the drug concentration. Therefore, x_b_ (equal to [ML]/[L]) can be obtained from Equation (A9) and R_obs_ can be expressed as follows (Equation (A10)):

R_obs_ = R_f_ + R_b_K[M_0_]/(1 + K[L]) (A10)

By plotting R_obs_ in the function of [M_0_], a straight line should be obtained; the slope of the line is the affinity index [A] (Equation (A11)):

[A] = KR_b_/(1 + K[L]) (A11)

which can be normalized to obtain [A^N^] (Equation (A12)).

[A^N^] = [A]/R_f_ = KR_b_/(R_f_(1 + K[L])) (A12)

For n binding sites of equal strength, the affinity index is (Equation (A13)):

[A] = KR_b_[L]^n−1^/(1 + K[L]^n^) (A13)

For n binding sites, each with a different thermodynamic equilibrium constant, the affinity index takes the form of Equation (A14):

(A14) $$\left[A\right]=\overset{n}{\underset{i}{\sum }}\left(\frac{n_{i}}{n_{t}}\frac{K_{i}R_{b_{i}}{\left[L\right]}^{n_{i}-1}}{1+K_{i}{\left[L\right]}^{n_{i}}}\right)$$

Independently from the complexation stoichiometry, a linear relationship always correlates the relaxation rate and the macromolecule concentration (Equations (A15) and (A16)):

R_obs_ = R_f_ + [A][M_0_] (A15)

and

ΔR/R_f_ = (R_obs_ − R_f_)/R_f_ = [A^N^][M_0_] (A16)

The linear fitting of the relaxation rates as a function of the macromolecule concentration [A^N^] can be obtained, providing a measure of ligand–macromolecule global affinity. The determination of this parameter represents a useful approach to compare the ability of different ligands to interact with the same macromolecule or to compare the ability of different macromolecules to bind the same drug.
