# Supplementary material for: NMR Investigation of the Interaction of Three Non-Steroidal Anti-Inflammatory Drugs with Human Serum Albumin
Source: Molecules. 2022 Oct 6;27(19):6647. doi: 10.3390/molecules27196647 (PMC9571845; doi:10.3390/molecules27196647)
Supplement: Supplementary file 1 [file molecules-27-06647-s001.zip › molecules-1918690-supplementary.pdf]

Supplementary Material

NMR Investigation of the Interaction of Three Non-Steroidal Anti-Inflammatory Drugs with Human Serum Albumin

Federica Aiello [1,*], Gloria Uccello-Barretta [2,*], Claudio Picchi [2], Samuele Nazzi [2], Alessandra Recchimurzo [2] and Federica Balzano [2]

[1] National Research Council, Institute for Chemical and Physical Processes (CNR-IPCF), Via G. Moruzzi, 1, 56124 Pisa, Italy

[2] Department of Chemistry and Industrial Chemistry, University of Pisa, Via G. Moruzzi 13, 56124 Pisa, Italy

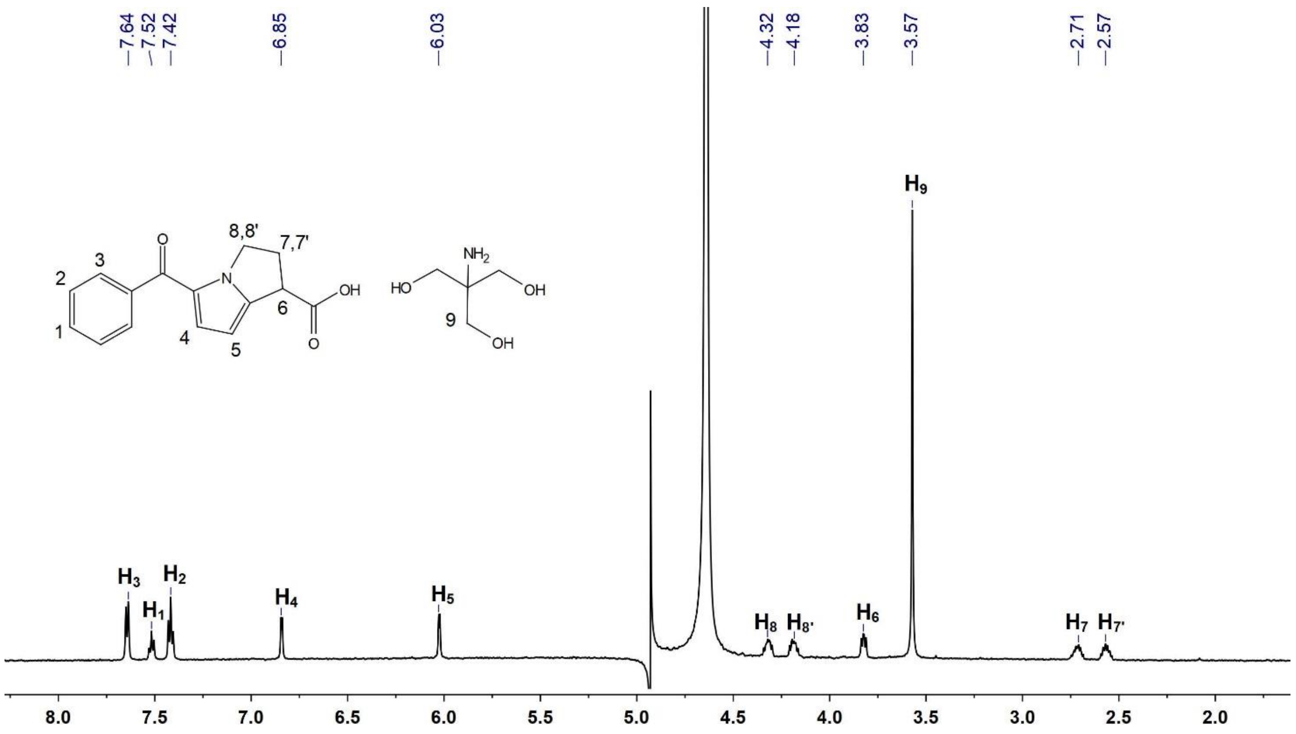

Figure S1. ^{1}H NMR (600 MHz, D_2O, pH 7.4, 298 K) spectrum of KTR (2 mM).

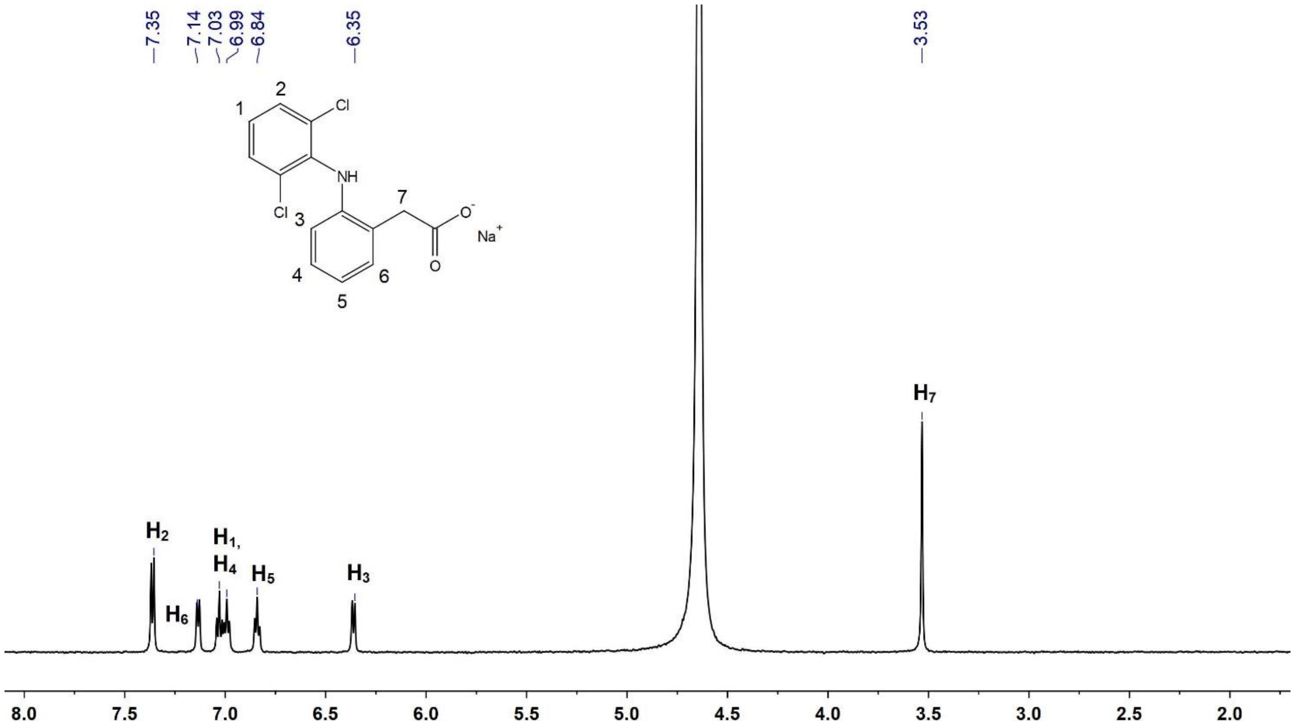

Figure S2. ^{1}H NMR (600 MHz, D_2O, pH 7.4, 298 K) spectrum of DCF (2 mM).

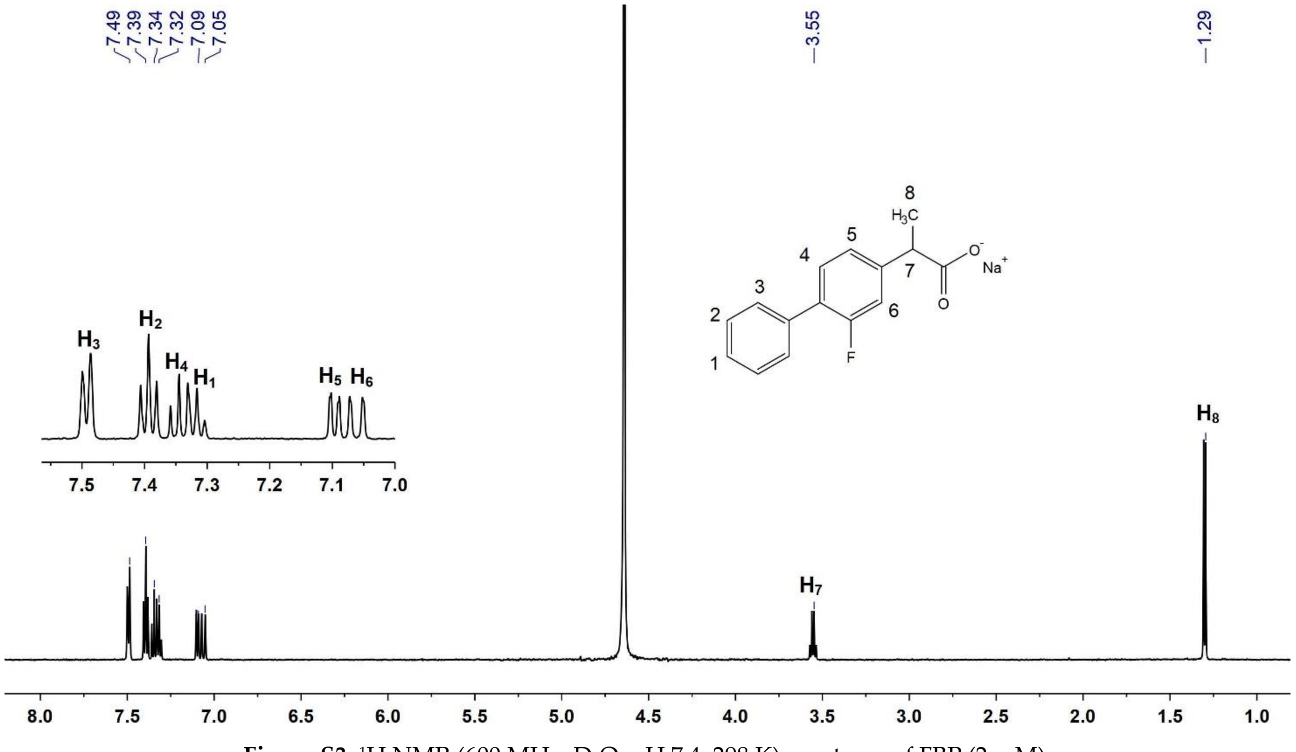

Figure S3. ^{1}H NMR (600 MHz, D_2O, pH 7.4, 298 K) spectrum of FBP (2 mM).

Table S1. ^{1}H mono-selective relaxation rates (R_1^{ms}, s^{-1}) (600 MHz, 25 °C, D_2O, pH = 7.4) of some protons of KTR (2 mM) alone and in mixtures with HSA at different molar ratios.

[HSA] (mg/mL)	H_1	H_4	H_5	H_6	H_7	$H_{7'}$	H_8	$H_{8'}$
	0.26	0.31	0.21	0.28	0.81	0.74	0.79	0.92
0.1	0.57	0.50	0.44	0.51	1.41	1.43	1.39	1.72
0.5	1.18	1.18	0.94	1.10	2.78	2.78	2.63	2.86
1.0	1.82	1.89	1.33	1.89	4.35	4.17	4.00	4.76
1.5	2.38	2.45	1.89	2.56	5.26	5.11	5.00	5.26

2.0	3.03	2.86	2.27	2.94	6.25	5.56	5.88	6.67

Table S2. Affinity indexes ([A^N], M^{-1}) and corresponding standard errors (ε, M^{-1}) calculated for KTR protons from NMR data.

proton	[A^N]	ε
H_1	321195	8006
H_4	268550	20519
H_5	297606	12924
H_6	306718	20595
H_7	205002	16434
$H_{7'}$	1953453	24511
H_8	196082	13144
$H_{8'}$	183820	17154

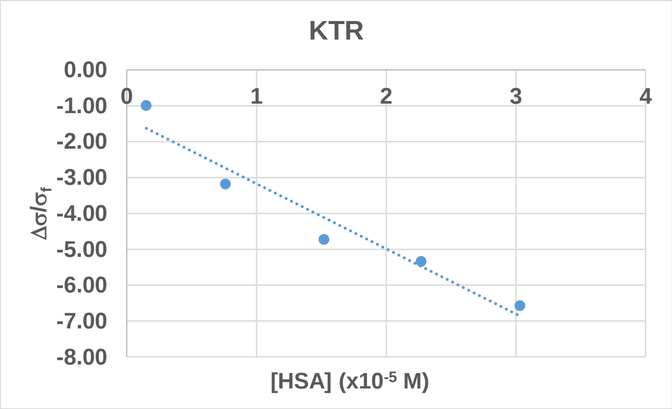

Figure S4. $\Delta\sigma/\sigma_f$ of KTR (2 mM) plotted in function of HSA concentration.

Table S3. ^{1}H mono-selective relaxation rates (R_1^{ms}, s^{-1}) (600 MHz, 25 °C, D_2O, pH = 7.4) of some protons of DCF (2 mM) alone and in mixtures with HSA at different molar ratios

[HSA] (mg/mL)	H_2	H_3	H_5	H_6	H_7
0	0.17	0.24	0.34	0.38	1.22
0.1	0.57	0.66	0.85	0.90	1.82
0.5	1.59	1.85	2.27	2.33	2.50
1.0	2.94	3.57	4.35	4.00	3.45
1.5	3.85	4.46	5.26	5.56	4.17
2.0	5.00	6.25	7.14	7.14	5.26

Table S4. Affinity indexes ([A^N], M^{-1}) and corresponding standard errors (ε, M^{-1}) calculated for DCF protons from NMR data.

proton	[A^N]	ε
H_2	903885	33206
H_3	803831	41542
H_5	624295	38067
H_6	563672	7629
H_7	96571	3173

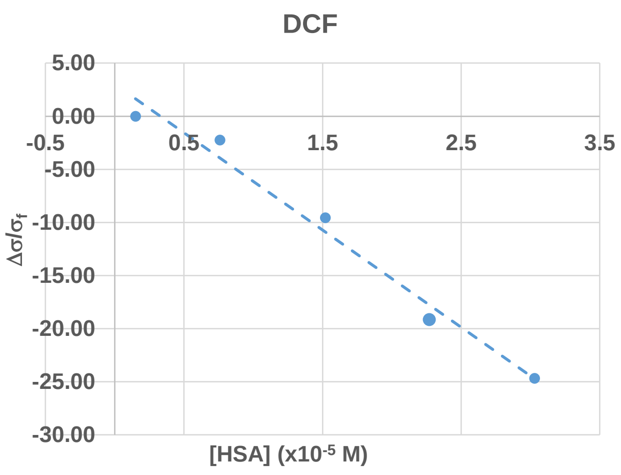

Figure S5. $\Delta\sigma/\sigma_f$ of DCF (2 mM) plotted in function of HSA concentration.

Table S5. ^{1}H mono-selective relaxation rates (R_1^{ms}, s^{-1}) (600 MHz, 25 °C, D_2O, pH = 7.4) of some protons of FBP (2 mM) alone and in mixtures with HSA at different molar ratios.

[HSA] (mg/mL).	H_3	H_7	H_8
0	0.34	0.34	1.33
0.1	0.58	0.57	1.45
0.5	1.39	1.37	1.89
1.0	2.50	2.27	2.22
1.5	2.94	2.81	2.63
2.0	3.33	3.85	3.45

Table S6. Affinity indexes ($[A^N]$, M^{-1}) and corresponding standard errors (ε, M^{-1}) calculated for FBP protons from NMR data.

proton	$[A^N]$	ε
H_3	281482	37266
H_7	327105	16837
H_8	48999	4822
